# Correction: Moradinezhad et al. Zein Multilayer Electrospun Nanofibers Contain Essential Oil: Release Kinetic, Functional Effectiveness, and Application to Fruit Preservation. *Foods* 2024, *13*, 700

**DOI:** 10.3390/foods15091470

**Published:** 2026-04-23

**Authors:** Farid Moradinezhad, Majid Aliabadi, Elham Ansarifar

**Affiliations:** 1 Department of Horticultural Science, Faculty of Agriculture, University of Birjand, Birjand 9717434765, Iran; fmoradinezhad@birjand.ac.ir; 2 Department of Chemical Engineering, Islamic Azad University, Birjand Branch, Birjand 9717711111, Iran; aliabadi@iaubir.ac.ir; 3 Department of Nutrition & Food Hygiene, Social Determinants of Health Research Center, School of Health, Birjand University of Medical Science, Birjand P.O. Box 97175-379, Iran

## Error in Figure

In the original publication [1], there was a mistake in Figure 1 as published. Figure 1 was inadvertently replaced with an incorrect image file. The corrected Figure 1. SEM images of Z (zein fiber without ZMEO), Z1 (one-layer zein fiber with ZMEO), Z3 (three-layer zein fiber with ZMEO), and Z5 (five-layer zein fiber with ZMEO). appear below.

## Error in Table

In the original publication [1], there was a mistake in Table 2 as published. There was a human data entry error in the transcription of the fitting parameters and associated numerical values. The corrected Table 2. The EE and kinetic model parameters for ZMEO release in zein multilayer fiber. appears below.

## Text Correction

There was an error in the original publication [1]. The text related to Table 2 in the manuscript. A correction has been made to 3.5. Encapsulation Efficiency (EE), Release Analysis, and Kinetic Modeling, Paragraph 2: The release profile of ZMEO from Z1 and Z3 films was fitted using the Korsmeyer–Peppas (R^2^ = 0.963, RMSE = 0.186) (R^2^ = 0.966, RMSE = 0.234) model, and the kinetic parameters were 0.429 and 0.406 (n) and 0.197 and 0.145 (k), respectively. This indicated that the release of ZMEO from Z1 and Z3 films was the mechanism of Fickian diffusion. The release kinetics for the multilayer (Z5) exhibited a good correlation with both the Higuchi (R^2^ = 0.981, RMSE = 2.121) and Korsmeyer–Peppas (R^2^ = 0.963, RMSE = 0.321) models.

The authors state that the scientific conclusions are unaffected. This correction was approved by the Academic Editor. The original publication has also been updated.

## Figures and Tables

**Figure 1:**
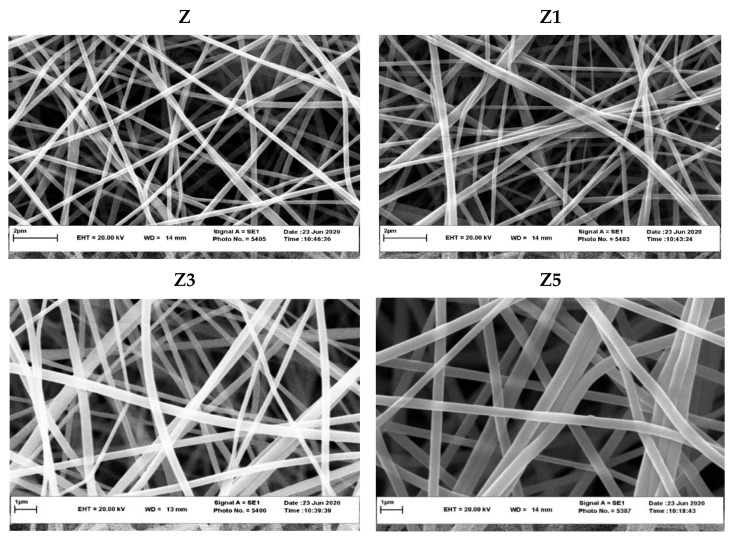
SEM images of Z (zein fiber without ZMEO), Z1 (one-layer zein fiber with ZMEO), Z3 (three-layer zein fiber with ZMEO), and Z5 (five-layer zein fiber with ZMEO).

**Table 2:** The EE and kinetic model parameters for ZMEO release in zein multilayer fiber.

Zein Multilayer	EE (%)	Model	R^2^	RMSE	Release Kinetic Model’s Data
Z1	43	Higuchi	0.796	2.813	k = 0.151
		Korsmeyer-Peppas	0.963	0.186	k = 0.197 n = 0.429
Z3	56	Higuchi	0.897	2.412	k = 0.099
		Korsmeyer-Peppas	0.966	0.234	k = 0.145 n = 0.406
Z5	82	Higuchi	0.981	2.121	k = 0.067
		Korsmeyer-Peppas	0.963	0.321	k = 0.026 n = 0.693
